# Hybrid Sequencing in Different Types of Goat Skeletal Muscles Reveals Genes Regulating Muscle Development and Meat Quality

**DOI:** 10.3390/ani11102906

**Published:** 2021-10-08

**Authors:** Yangyang Pan, Sijia Chen, Shu Niu, Xilin Bi, Liying Qiao, Kaijie Yang, Jianhua Liu, Wenzhong Liu

**Affiliations:** 1 Department of Animal Genetics, Breeding and Reproduction, College of Animal Science, Shanxi Agricultural University, Taigu 030801, China; panyy@sxau.edu.cn (Y.P.); chensijia8297@163.com (S.C.); niushu1387@163.com (S.N.); bixilin9645@163.com (X.B.); liyingqiao1970@163.com (L.Q.); kjyang@sxau.edu.cn (K.Y.); ljhbeth@163.com (J.L.); 2 Department of Fundamental Veterinary Science, College of Veterinary Medicine, Shanxi Agricultural University, Taigu 030801, China

**Keywords:** skeletal muscle, growth, meat quality, full-length transcriptome, hybrid sequencing, goat

## Abstract

**Simple Summary:**

Skeletal muscle development and meat quality are key traits of considerable importance to consumers and farmers. In the past, second-generation sequencing technology has been used to study genes regulating muscle development and meat quality. However, with the limitation of read length, goat transcriptome was constructed mainly on the basis of the merging of short reads, resulting in an insufficient understanding of goat transcriptome structures. Identification of full-length transcript structure was still a challenge. Therefore, in this study, a hybrid sequencing was conducted that combined the long-reading character of third-generation sequencing with the quantification ability of second-generation sequencing. By comparing the longissimus dorsi and biceps femoris muscles, genes and transcript isoforms regulating meat quality and muscle development of goat were identified. A large number of novel loci and isoforms were identified in the goats. Functional annotation of these genes showed that they were associated with skeletal muscle development and lipid metabolism.

**Abstract:**

Domestic goats are commonly reared for meat and milk production in several regions of the world. However, the genetic mechanism underlying muscle development and meat quality of goats is limited. Therefore, the aim of this study was to identify known and novel genes regulating muscle development and meat quality of goats using second- and third-generation sequencing technologies. To achieve this, the meat quality and transcriptomes of longissimus dorsi (LD) and biceps femoris (BF) muscle tissues of Lingqiu Greyback goats were examined and compared. Differentially expressed genes (DEGs) and isoforms (DEIs) were functionally annotated. Results showed that 45,574 full-length transcripts covering 18,491 loci were characterized, and 12,566 genes were co-expressed in all samples. Differential expression analysis identified 231 DEGs, including 45 novel genes in the LD and BF muscles of the goats. Additionally, 1173 DEIs were found, in which 642 novel isoforms were identified in this study. Functional annotation and pathway analysis of the DEGs and DEIs revealed that some of them were associated with muscle growth and lipid metabolism. Overall, the findings of this study contribute to the understanding of the transcriptomic diversity underlying meat quality and muscle development of goat.

## 1. Introduction

Goats (*Capra hircus*) are reared in several countries, especially in mountainous regions and developing countries in subtropical and arid regions, for their meat, milk, and wool. Additionally, goats possess high adaptability to harsh environmental conditions [1] and are consumed for their flavor and palatability. Among several economic traits, meat yield and quality are key traits of considerable importance to farmers and consumers; however, they are controlled by both genetic and environmental factors [2]. Over the years, several studies have attempted to improve the understanding of the molecular and genetic mechanisms associated with muscle growth and meat quality, leading to improved meat quality and yield of some domestic animals [3]. Some functional genes and pathways, such as myostatin [4], muscle regulatory factors [5], insulin-like growth factors [6], and mitogen-activated protein kinase (MAPK) [7], have been found to play crucial roles in myocyte proliferation and differentiation. Compared with meat from other domestic animals, such as sheep, goat meat possesses higher dietetic qualities [8], such as leaner carcass and lower fat and intramuscular fat content [9]. However, the growth rate of goat muscle is usually lower than that of sheep. Meat tenderness is another quality trait of importance to consumers [10]; however, the level of tenderness differs considerably between muscle types [11]. For instance, it has been reported that the biceps femoris (BF) is tougher than the longissimus dorsi (LD) muscle in cattle [12].

Additionally, consumers have a higher preference for meat from the dorsal and hind limbs of goat [10]. Therefore, comparative examination of different muscles can help elucidate the genes and signaling pathways regulating myogenesis and meat quality traits. Previous studies have examined transcriptional level changes in goat LD at different postnatal stages [13,14]. However, these studies were mainly based on second-generation sequencing, which is characterized by short read length and is primarily effective in identifying gene expression levels. The understanding of goat transcriptome, as well as identification of novel isoforms and evolutionary events, such as alternative splicing, is limited compared with model animals, such as mice. Moreover, research on genetic factors associated with goat skeletal muscle development and quality and the underlying mechanisms are limited. However, with the advancement of third-generation sequencing technology, full-length RNA sequencing is possible, making it easier to identify and annotate novel genes [15]. Additionally, a combination of short-read and full-length sequencing could help identify more loci and isoforms.

Therefore, the aim of this study was to identify genes regulating muscle quality and yield in Lingqiu Greyback goats using full-length and short-read sequence data of the BF and LD muscles. Phenotypes such as meat tenderness, color, and marbling score of BF and LD were compared. Genes and isoforms in these two types of skeletal muscles were identified and their expressions were quantified. Functions of differentially expressed genes (DEGs) and isoforms (DEIs) were annotated, revealing genes associated with muscle development and meat quality. The present study will benefit future investigations of the mechanism underlying goat muscle growth in the development of intramuscular fat, flavor, tenderness, and toughness.

## 2. Materials and Methods

### 2.1. Animal Management and Sample Collection

Four Lingqiu Greyback (male) goats were randomly selected and individually housed in stalls under similar environmental conditions and fed the same diet. Composition of diets is shown in the Table S1. The goats were exsanguinated at 10 months of age in an aseptic environment, and the carcasses were bisected at the last rib perpendicular to the *longissimus thoracis* muscle. Digital photos of the loin area were captured, and the loin area was measured using ScanStar K software (Matthaus, Klausa, Germany). Rib thickness was measured between the 12th and 13th ribs of the carcass. LD and BF muscle tissues from the left side were collected using RNase-free surgical instruments. One portion of the LD and BF samples was cut into approximately 100 g pieces, snap-frozen in liquid nitrogen, and stored in a freezer at −80 °C for total RNA extraction. The other portion of the samples and the remaining carcasses were chilled at 4 °C for meat quality analysis.

### 2.2. Meat Quality Analysis

Meat quality analysis was conducted according to previously reported methods [16,17]. Briefly, the pH of the LD and BF muscles was measured after 45 min and 24 h of chilling using a pH-STAT meter (SFK-Technology, Hvidovre, Denmark). Additionally, meat color, water loss, water holding capacity, and shear force (tenderness) of the samples were measured. Meat color (L, a, and b) was measured using a spectrophotometer (Konica Minolta Inc., Tokyo, Japan). For the water loss ratio, 1 cm thick steaks (area 5 cm^2^) were cut from the LD and BF and covered with filter papers. The weight of the samples was calibrated before and after putting 35 kg of pressure. Water-holding capacity was analyzed on the basis of the weight before and after cooking. A 2000 W steam-oven was used to cook the meat (100 g) for 45 min, and the weight was determined after 30 min of cooling at 20 °C. For shear force measurement, 2.54 cm thick meat was cooked in an oven at 177 °C. Once the center temperature of the meat reached 70 °C, the samples were taken out and cooled to room temperature. After further cooling at 4 °C overnight, the maximum shear force was determined using a shear force instrument (Mecmesin, Horsham, UK).

### 2.3. Short-Read RNA Sequencing

LD and BF samples from the four goats were ground in liquid nitrogen to avoid RNase. RNA was extracted using TRIzol kit (Takara, Dalian, China), according to the manufacturer’s instructions, and digested with DNase I (RNase-free). mRNA was enriched using magnetic oligomeric (dT) beads, cut into short fragments using fragmentation buffer, and reverse transcribed into cDNA using random primers, which was used as a template for the synthesis of first- and second-strand cDNA. Thereafter, short cDNA fragments were purified and used for sticky end repair. The 3′ ends of the cDNA were added to base A and connected with adaptors. Suitable fragments were selected on the basis of their size after agarose gel electrophoresis and were used as templates for PCR amplification. After cDNA amplification, RNA quality and quantity were assessed using Agilent 2100 Bioanalyzer and ABI StepOnePlus Real-time PCR System. The sequencing of eight qualified libraries was performed by BGI (Shenzhen, China) on an Illumina platform using Illumina Hiseq^TM^ 2000.

### 2.4. Full-Length RNA Sequencing

LD and BF muscle tissues from four goats were pooled together in equal proportions. The mixed sample was lysed using RNAiso Plus (Takara, Dalian, China), and total RNA was extracted using TRIzol kit, according to the manufacturer’s guidelines. RNA degradation and contamination were monitored using 1% agarose gel. RNA concentration and purity were assessed using Nanodrop 2000 spectrophotometer (Thermo Fisher Scientific, Waltham, MA, USA), and RNA integrity was assessed using Agilent 2100 Bioanalyzer (Agilent Technologies, Santa Clara, CA, USA). The concentration of mixed RNA was over 200 ng/mL, and the total weight was over 1 μg. cDNA was synthesized from mRNA using a SMARTerTM PCR cDNA Synthesis Kit (Clontech, San Jose, CA, USA), according to the manufacturer’s instructions. Briefly, oligo dT primers were used to synthesize first-chain cDNA by pairing with the mRNA polyA tail. cDNA was amplified by large-scale PCR and purified using AMPure PB beads (Pacific Biosciences, Menlo Park, CA, USA), and fragments smaller than 1 kb were removed. SMRT adaptors were aligned to cDNA after end repair, and exonucleases were used to digest fragments without the adaptors. The BluePippin system (Sage Science, Beverly, MA, USA) was used to determine the size of cDNA fragments. Three shotgun libraries of different lengths (1–2 kb, 2–3 kb, and 3–6 kb) were obtained. Full-length transcriptome sequencing was conducted by Macrogen (Shenzhen, China) using PacBio RS II (Pacific Biosciences, Menlo Park, CA, USA). Each shotgun library was sequenced using two SMRT cells.

### 2.5. Analysis of Short-Read Sequencing Data

High-throughput sequencing data were analyzed using a previously reported method [18]. Briefly, raw “reads” were filtered to obtain high quality transcriptome sequence data. First, all reads with adaptor contamination were discarded. Second, reads with unknown nucleotides (*n*) comprising more than 10% were removed. Third, low-quality reads with percentage of low-quality bases over 50% were discarded. After quality control using FastQC (https://www.bioinformatics.babraham.ac.uk/projects/fastqc/, accessed on 8 March 2021), clean reads were mapped to the *Capra hircus* reference genome (ARS1) using Hisat2 [19] and mapped to a gene reference using Bowtie2 (http://bowtie-bio.sourceforge.net/index.shtml, accessed on 8 March 2021). Expectation Maximization (RSEM) software was used to assemble the transcripts and to estimate their expression levels, normalized as fragments per kilobases per million (FPKM) [20]. Additionally, to ensure that the present data were replicable, we performed correlation analysis of the gene expression levels of the samples [21]. Pearson’s correlation was performed on the basis of FPKM of genes using plotCorrelation, a tool in deepTools2 [22]. The coefficient (R^2^) was plotted as heatmap. Thereafter, differential expression analysis was performed to determine differentially expressed genes (DEGs) using DESeq2 [23]. In this study, paired nature of samples was not considered and only differences between LD and BF groups were provided by DESeq2. Genes with FPKM > 0, FDR < 0.05, and|log2(fold change (BF/LD)) > 1 were considered differentially expressed. Gene Ontology (GO, http://geneontology.org, accessed on 24 March 2021; FDR ≤ 0.05) and Kyoto Encyclopedia of Genes and Genomes (KEGG; Q-value between 0 and 1) enrichment for DEGs that had FPKM ≥ 1 in all samples were performed using the OmicShare tools, a free online platform for data analysis (www.omicshare.com/tools, accessed on 24 March 2021).

### 2.6. Analysis of Full-Length Sequencing Raw Data

Polymerase reads obtained from PacBio RS II system were analyzed using SMRT Link v7.0.1 (Pacific Biosciences, Menlo Park, CA, USA), according to the IsoSeq^TM^ protocol (Pacific Bioscience, Menlo Park, CA, USA). Sequencing adaptors were removed from the polymerase reads to form the subreads. Circular consensus sequences (CCSs) were processed from subreads (Parameters: minimum number of passes = 3, minimum predicted accuracy = 0.99). To produce draft transcripts (parameter: minimum read length = 50, maximum read length = 15,000), we removed 5′ and 3′ ends from the cDNA primers. CCS refers to the sequence of the same zero-mode waveguide well with more than two sequencing times, and the sequence error is corrected by the multiple sequencing results; the accuracy is a low-error-rate sequence that meets a fixed value or more [24]. Two types of CCSs, full-length (FL) and full-length non concatemer (FLNC), can be identified in the full-length transcriptome sequencing. FLNCs containing polyA were used for the subsequent analysis. Since SMRT sequencing has a much higher error rate than Illumina high-throughput sequencing (short-read sequencing), self-correction using multiple sequencing results and short-read sequencing was employed in the present study. Reads obtained from short-read sequencing in LD and BF muscle samples was used to correct the full-length sequencing errors using LoRDEC software [25]. Raw FLNC and error corrected FLNC sequences were mapped to the goat reference genome (ARS1, RefSeq assembly accession GCF_001704415.1) to count the global and local percentage of identity (PID) using GMAP [26]. According to the genome mapping results, FLNCs with higher PID remained and were classified into four types: unmapped, multiple-best, low PID, and high quality. In order for the transcript alignment position of the same loci and the transcript alignment position of different loci to be distinguished, transcripts were the same loci when they had the same alignment direction, and the area overlap between the alignment start sites reached 20%, with there being at least one exon overlap at the same time of more than 20%. To identify the isoform from the same loci, we removed redundant and low-quality isoforms. When isoforms had identical splicing sites, the shorter one was removed. If the isoforms obtained after data analysis contained the first splice donor site of annotated isoforms, these isoforms were considered full-length isoforms, and the corresponding FLNCs were considered full-length FLNCs.

### 2.7. Identification and Functional Annotation of Novel Genes and Isoforms

After comparison with the reference genome, novel genes were identified from the full-length transcript sequencing data. Genes that did not overlap with annotated genes, or when the overlap was less than 20%, or when the overlap was over 20% but had a converse direction with the annotated genes, were considered novel genes [27]. Isoform identified in this study was considered as a novel isoform when it had one or more novel splicing sites or was not the same single exon when compared with the reference transcript. Each novel isoform was supported by at least two FLNCs, or one FLNC read with PID higher than 99.

To obtain a comprehensive functional annotation of the isoforms, we mapped genes to several databases, including the National Center for Biotechnology Information (NCBI) non-redundant protein sequence (NR), GO [28], complete eukaryotic genomes (KOG) [29], KEGG [30], KEGG Orthology (KO) [31], and Swiss-Prot [32], using Diamond [33].

### 2.8. Identification of lncRNAs and Novel Isoforms’ Open Reading Frames

Isoform sequences of known and novel genes obtained from full-length sequencing were mapped to the NR, KOG, KO, and Swiss-Prot databases in order to filter out the coding sequences. The protein-coding probability of the remaining sequences was estimated using CPAT [34] with default parameters. Isoforms with length of ≥200 nt or coding probability of ≤0.5 were considered as lncRNAs. Alternative splicing (AS) events were identified by comparing different isoforms of the same gene using Astalavista [35]. FLNCs were aligned to the reference genome, and fusion genes were predicted using fusion gene prediction software (Frasergen, Wuhan, China). Partner genes of each fusion gene were represented as 5′ and 3′ sequences, respectively. On the basis of the aligned location of FLNC 5′ sequences in the genome, we identified a reliable alternative polyadenylation of RNA (APA) using Tapis [36]. APA supported by at least two FLNCs was maintained.

Open reading frames (ORFs) of novel isoforms, except the lncRNAs, were predicted using TransDecoder (https://github.com/TransDecoder/TransDecoder/wiki, accessed on 12 March 2021). ORFs that code amino acids with length ≥ 100 were aligned with the protein sequences in the Swiss-Port database to identify homologous proteins using BlastP. Hmmscan [37] was used to scan Pfam [38] database to identify the structural domains of the proteins. The ‘Predict’ function of TransDecoder was used to evaluate the predicted ORFs on the basis of their homologous and structural domains.

### 2.9. Quantitative Real-Time PCR (qPCR) and Data Analysis

The relative expression of DEGs identified by RNA-seq was verified using qPCR. Briefly, RNA was extracted from the samples using TRIzol kit, according to the manufacturer’s instructions. cDNA from 1 μg of RNA from each sample was synthesized using PrimeScript^TM^ RT Reagent Kit with gDNA Eraser (RR047A, Takara, Dalian, China). qPCR was conducted with TB Green^®^ Premix Ex Taq™ II (Tli RNaseH Plus, RR820A, Takara, Dalian, China) using CFX Connect Real-Time PCR Detection System (Bio-Rad, Hercules, CA, USA). Thirteen genes with FRKM > 50 were randomly selected. Primers were designed using Primer-BLAST (https://www.ncbi.nlm.nih.gov/tools/primer-blast/, accessed on 4 April 2021). The list of qPCR primers used in this study is found in Table S2.

Data from three replicates of each sample were collected and mRNA expression of samples was normalized to that of the reference gene GAPDH, and fold change was calculated using the 2^−ΔΔCT^ method. All data obtained from the qPCR in the present study were analyzed using one-way analysis of variance, and Duncan’s multiple range test was used for post hoc analysis using Prism 8 (GraphPad Software, San Diego, CA, USA).

## 3. Results

### 3.1. Slaughter Performance and Meat Quality

Body weight was measured before slaughter. As shown in Table S3, the average body weight and mean carcass weight of the 10-month-old goats used in the present study were approximately 28 kg and 12 kg, respectively, indicating that the goats had proper growth rates according to the Agricultural Industry Standard of China (NY/T 630-2002). Slaughter performance parameters, such as fur, head, feet, blood, internal organs, and bone weights were also measured. Rib thickness, indicating the fat content of carcasses, showed that the goats were slim.

Additionally, meat quality parameters of the LD and BF muscles were analyzed after slaughter. As shown in Table 1, there were no significant differences in the pH values of the LD and BF muscles at 45 min and 24 h postmortem. Similarly, there were no significant differences in the lightness and yellowness of two muscle types; however, the redness of BF muscle was significantly more intense (*p* < 0.05) than that of the LD muscle. Although there were no significant differences in water loss and water holding capacity of the LD and BF muscles, the LD muscles had significantly lower (*p* < 0.05) shear force and higher (*p* < 0.05) marbling score than BF.

### 3.2. Transcriptome Profiling of Goat Longissimus Dorsi and Biceps Femoris Muscles

**Figure 1:**
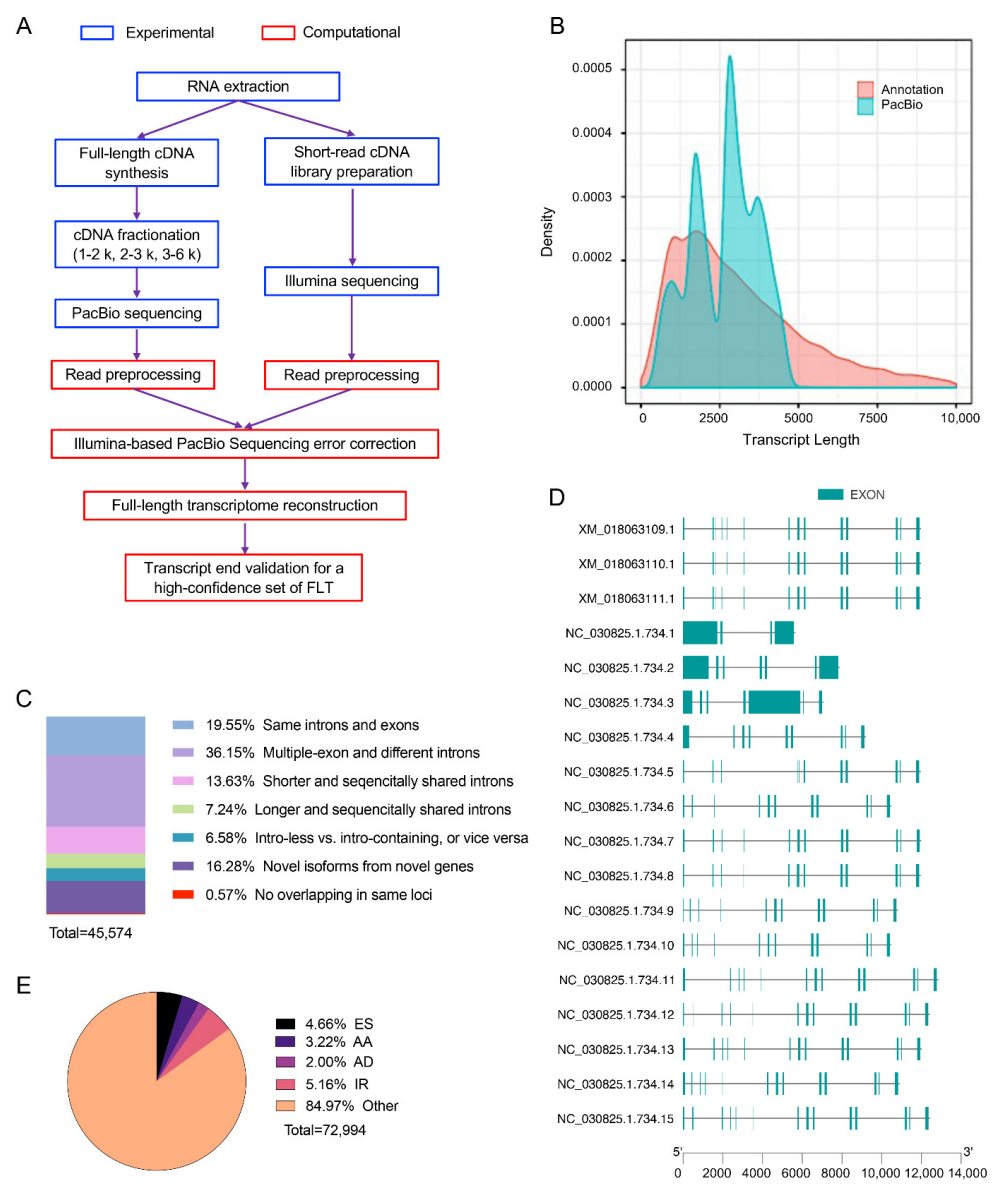
Full-length transcriptomic sequencing improves the understanding for goat transcriptome. (**A**) Hybrid sequencing strategy used in this study. PacBio third generation and Illumina second generation sequencing were used to obtain the full-length and expression level of transcript, respectively. (**B**) Comparison between goat RefSeq from NCBI and PacBio data obtained from this study. (**C**) Classification of full-length transcripts obtained from third generation sequencing. (**D**) Known transcripts (XM_*) of TNNT1 already annotated by NCBI and newly discovered transcripts (NC_*) in this study. (**E**) Types of alternative splicing identified from full-length sequencing. ES, exon skipping; AA, alternative acceptor; AD, alternative donor; IR, intron retention.

Next-generation high-throughput RNA sequencing and SMRT Iso-Seq were performed to comprehensively examine the transcriptome of goat LD and BF muscle tissues (Figure 1A). A total of eight cDNA RNA-seq libraries were generated, four libraries from LD and BF muscle tissue each. The libraries were subjected to Illumina short-read sequencing. After quality filtering, 196,018,497 clean reads (over 99%) were obtained from the reads (Table S4). The clean reads were mapped to the reference goat genome. Approximately 93.52% (SEM = 0.27%) clean reads from each library were mapped to the genome (Table S5), and 85.33% (SEM = 0.40%) of clean reads were annotated in the combined transcript library containing known transcripts and novel transcripts identified by full-length sequencing (Table S6).

Full-length cDNA sequences are helpful in annotating and identifying authentic transcripts from animal tissues. In the present study, RNAs from the LD and BF muscles from four individuals were equally mixed for PacBio SMRT sequencing, which provides single-molecule, real-time, and full-length sequences. After removing adapter dimers (0–10 bp in length) and short inserts (11–100 bp in length), 144,625 (1–2 kb), 133,687 (2–3 kb), and 135,310 (3–6 kb) CCSs were obtained from different fragmented libraries (Table 2). After error correction using short reads from RNA-seq, 225,417 high-quality FLNC reads with average length of 2138 bp were obtained, among which 94.49% were mapped to the reference genome (Table 3). A total 18,491 loci and 45,574 isoforms were identified from the FLNCs (Table 4). Although the PacBio FLNCs had fewer short loci (<2 K in length) than the RefSeq, they had longer loci (>2 K in length) (Figure 1B). The total loci identified in the present study covered 81.93% of the 22,570 loci in goat RefSeq. Full length ratios of isoforms and FLNCs were also evaluated, and it was found that 72.43% multi-exon isoforms (34,833 in total) and 90.50% multi-exon FLNC reads (153,547 in total) were full-length.

### 3.3. Characterization of Novel Isoforms

One of the advantages of full-length transcriptome sequencing is that the sequence does not need to be assembled; thus, the gene models and transcribed genes could be fully annotated. After comparison with the goat RefSeq annotation, full-length transcripts were classified (Figure 1C). A total of 6966 novel genes containing 7420 isoforms were identified, as they did not overlap with any annotated genes in goat RefSeq. Overall, 9289 genes containing 29,246 new isoforms were annotated in RefSeq. For example, only three transcripts of TNNT1 were annotated in the goat RefSeq, and 15 new transcripts were identified by full-length sequencing (Figure 1D).

The novel isoforms (7420) were aligning against the NR, GO, KOG, KEGG, and Swiss-Prot databases for functional annotation. Results showed that 72.79%, 29.84%, 49.68%, 4.15%, and 25.22% of the novel isoforms were mapped to the NR, GO, KO, KOG, and Swiss-Prot databases, respectively; however, 26.91% of the novel isoforms were not annotated in any of the databases. KOG, GO, and KEGG annotations of the novel isoforms are shown in Figures S1–S3, respectively. Novel isoforms from novel and known genes were mapped to the NR, KO, KOG, and Swiss-Prot databases, followed by prediction of coding capacity. After filtering, 2745 long non-coding RNAs (lncRNAs) with low coding capacity and/or were more than 200 bp in length, were identified (Table S7), highlighting the advantage of SMRT sequencing in identifying lncRNAs.

### 3.4. Alternative Splicing Events Analysis

In the present study, full-length transcripts were analyzed for alternative splicing (AS) events. Overall, 72,994 AS events including exon skipping (ES), alternative acceptor sites (AA), alternative donor sites (AD), and intron retention (IR) were identified (Figure 1E). ES, AA, AD, and IR events occurred in 3617 genes, among which 49 were DEGs. The IR is an alternative splicing mode that introns rather than exons being spliced out, are retained in mature mRNAs. The percentage of IR events in all AS events was lower in the BF muscle (32.98%) than that in the LD muscle (34.23%).

### 3.5. Overall Gene Expression Level

**Figure 2:**
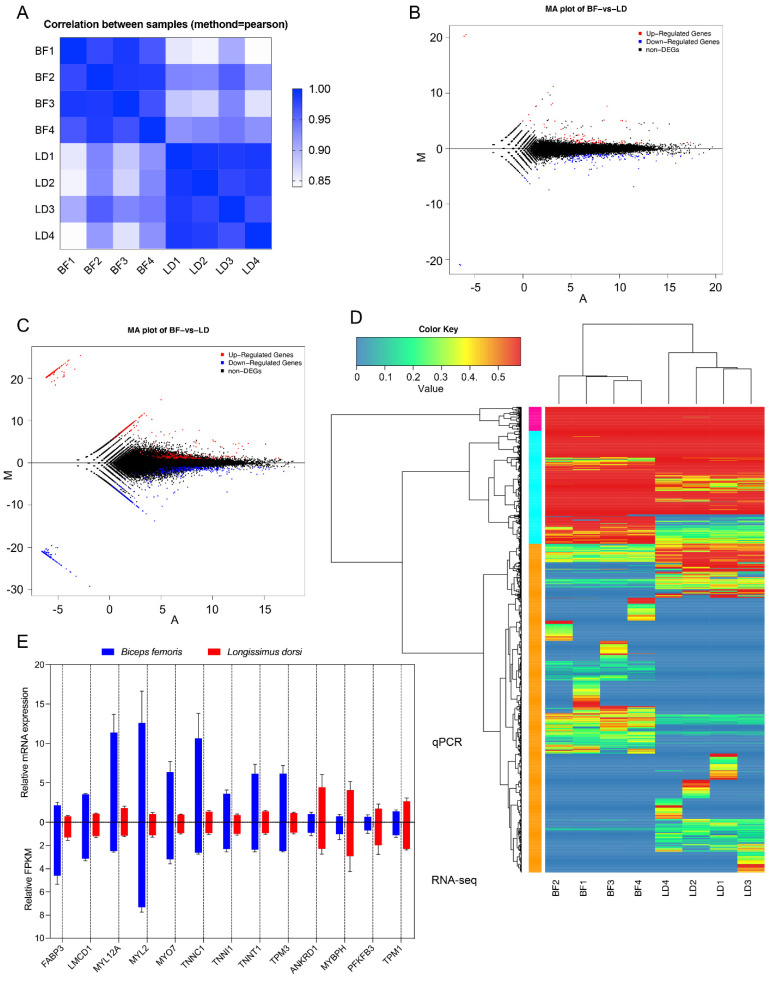
Short-read sequencing quantifies the expression of genes and transcripts of goat muscles. (**A**) Correlations between eight samples used for short-read sequencing. The correlation between each pair of samples in the sequencing libraries was calculated from the FPKM value. Samples with a Pearson’s correlation value ≥ 0.92 were considered as the same experimental replicates. The deeper blue color represents closer correlation between samples. (**B**) MA plot indicating differentially expressed genes between the BF and LD muscles. The red and blue dots represent the upregulated and downregulated genes in BF compared to LD muscles, respectively. |log2(FoldChange)| > 1 and padj < 0.05 is considered up- or downregulated. (**C**) MA plot indicating the differentially expressed isoforms between the BF and LD muscles. (**D**) Cluster enrichment of differentially expressed transcripts from LD and BF samples. The gradient color key represents the adjusted *p*-value, which is more significantly different when having more red color. (**E**) RNA-seq results are consistent with qPCR data. qPCR was used to verify the expression profiles of 13 randomly selected differentially expressed genes in RNA-seq results. The gene expression levels obtained from qPCR were normalized to GAPDH and are shown as mean ± standard error of mean (SEM) (*n* = 4). Data from RNA-seq is shown as mean FPKM ± SEM (*n* = 4).

Exon coverage of the eight libraries was calculated as the percentage of exons covered by the reads. In all the libraries, over 87% of the exons had 90–100% coverage (Table S3). The expression level of each gene and transcript was normalized as FPKM. Statistics of the number of genes and isoforms at different expression levels shown as FPKM are shown in Tables S8 and S9. A total of 19,021 genes and 36,066 isoforms were identified in all of the BF samples, among which 1059 genes and 4625 isoforms were unique to the BF samples. Additionally, 18,668 genes and 35,481 isoforms were identified in all the LD samples, among which 703 genes and 4059 isoforms were unique to the LD samples. Correlation analysis of the gene expression profiles of the different samples showed that the gene expression profiles in the same type of tissues were highly correlated (Figure 2A). To compare the ability of identifying isoforms between full-length and short-length sequencing, we analyzed the shared and exclusively identified isoforms. Among the 53,128 isoforms that identified from the hybrid sequencing strategy, 11,422 isoforms were commonly identified by both PacBio and Illumina sequencing, 34,152 isoforms were exclusively identified by PacBio platform, and 7554 isoforms were exclusively in the Illumina data.

### 3.6. Differentially Expressed Genes and Isoforms in Different Muscles

To further examine the differences in gene expression patterns between the two muscle types, we analyzed the comparative transcriptome. Already annotated and novel genes and isoforms with FPKM > 0 were used in the calculation. Data from the same tissue types were also grouped. Overall, a total of 231 DEGs (|log2(FoldChange)| > 1 & padj < 0.05), including 45 novel genes, were identified in the BF and LD muscles (Figure 2B and Table S10), among which 147 were downregulated, whereas 84 were upregulated. Additionally, 1173 differentially expressed isoforms (DEI; |log2(FoldChange)| > 1 & padj < 0.05), including 642 novel DEI, were identified in the BF and LD muscles, among which 534 were upregulated and 639 were downregulated (Figure 2C and Table S11). The ability of identifying DEGs and DEIs between hybrid sequencing strategy and only using short-length sequencing was compared. Results showed that 46 DEGs were commonly identified by two methodologies, 185 DEGs were exclusively identified by the hybrid sequencing strategy, and 6 DEGs were exclusively identified by only using the Illumina sequencing. Regarding DEIs, no DEIs were shared by the two methodologies, 1173 DEIs were exclusively identified by the hybrid sequencing strategy, and 94 DEIs were exclusively identified by the Illumina-only sequencing. Cluster analysis can help visualize the levels of gene expression and expression patterns in multiple samples. Cluster analysis revealed that the same type of muscle had highly similar gene expression levels and patterns among different individuals, while different types of muscles had distinct patterns (Figure 2D).

To further validate the accuracy of the RNA-sequencing and data analysis procedures, which might introduce errors, we performed qPCR of the samples to examine the expression profiles of 13 DEGs (nine upregulated and four downregulated DEGs). The relative expression of the genes was similar to that of the RNA-seq and significantly expressed (*p* < 0.05) between LD and BF, thus validating the RNA-seq data (Figure 2E).

**Figure 3:**
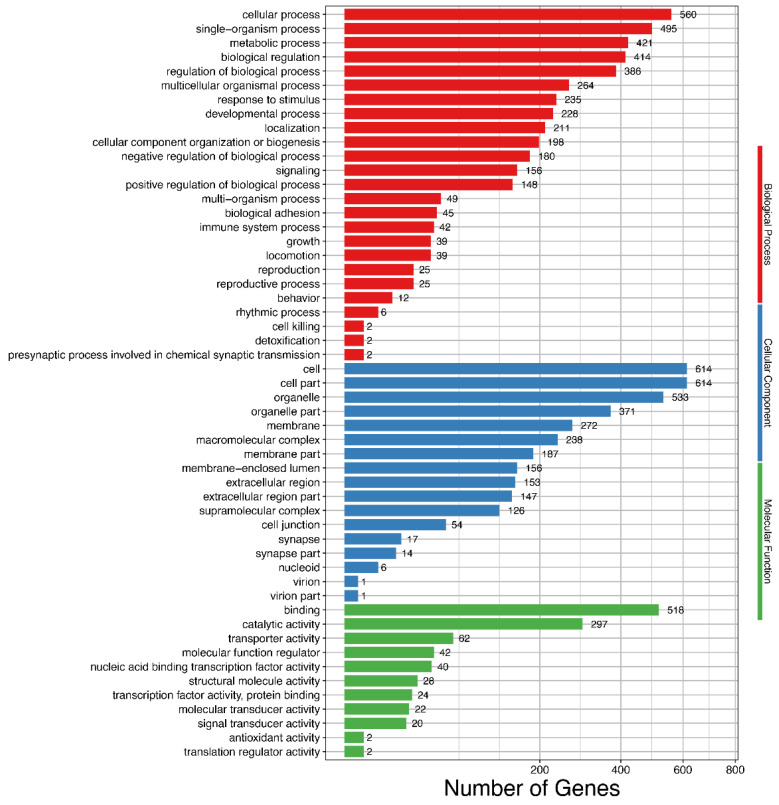
Gene Ontology (GO) enrichment of differentially expressed isoforms. Hyper-geometric distribution method is used. FDR ≤ 0.05 is considered as significantly enriched.

Functional annotation of the DEIs in GO database showed that they were enriched in molecular functions, cellular components, and biological processes (Figure 3 and Table S12). Among the molecular function terms, the DEIs were mainly enriched in binding functions such as cytoskeletal protein binding, metal ion binding, and RNA binding. For cellular component, 115 isoforms were enriched in myofibril. For the biological processes, the DEIs were enriched in developmental processes such as muscle structure development; muscle tissue and organ development and muscle cell development; and metabolic processes, such as glycerolipid metabolism, carbohydrate metabolism, neutral lipid metabolism, and fatty acid metabolism. Additionally, functional annotation of the DEGs using GO database showed that the DEGs were significantly enriched in 24 GO terms involved in biological processes of skeletal muscle growth and meat quality (Table 5). The roles of these genes were highly correlated with muscle tissue and fiber development, slow/fast-twitch skeletal fiber transition, fat development and metabolism, and collagen formation.

**Figure 4:**
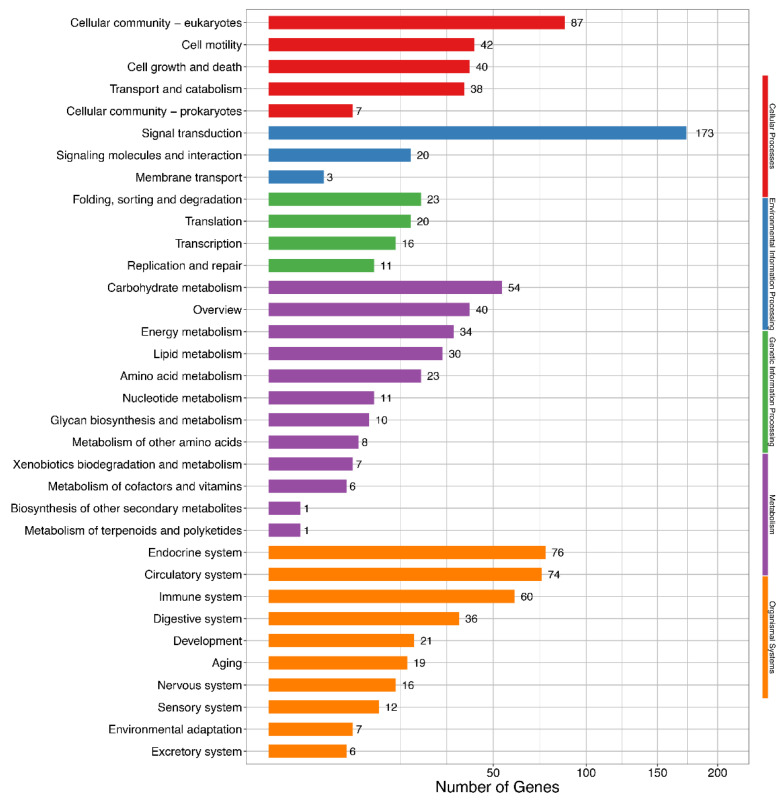
Kyoto Encyclopedia of Genes and Genomes (KEGG) enrichment of differentially expressed isoforms. Hyper-geometric distribution method is used. FDR ≤ 0.05 is considered as significantly enriched.

KEGG pathway analysis showed that 30 DEIs were enriched in the lipid metabolism pathway (Figure 4 and Table S13) involved in glycerolipid metabolism, synthesis and degradation of ketone bodies, fatty acid elongation, unsaturated fatty acids biosynthesis, and sphingolipid metabolism.

## 4. Discussion

In the present study, second- and third-generation sequencing technologies were employed to examine the transcriptome of the LD and BF muscles of goat to determine genes regulating muscle development and meat quality. Identified genes and isoforms were annotated against GO and KEGG databases. Over 85% clean reads were mapped to a merged transcripts pool that contained the known transcripts from NCBI and new transcripts from the full-length sequencing that conducted in this study. The clean reads that could not mapped to this merged pool theoretically presented the exclusive transcripts that identified by RNA-seq. Additionally, 7554 isoforms were specifically identified by RNA-seq, indicating that the short-read sequencing still have the advantages of high-depth and low-cost. A deeper full-length sequencing combined with short-length sequencing might be needed in the future to increase the capture rate. However, there were still 6966 novel loci yet to be annotated in the reference genome, and 36,666 novel isoforms from novel or known loci were identified in this study, thus improving understanding of skeletal muscle-related genes in transcriptome of goats. The length and quantity of transcripts identified in the present study indicated that the hybrid sequencing strategy employed improved goat genome annotation. However, although the average length of transcripts identified in this study was longer than that of the reference annotation, our approach did not cover extremely long transcripts in full-length forms. Compared with the reference annotation, transcripts longer than 5000 nt were covered with much lower densities, which may be because the fragmentation step used in the library preparation step limited the reading of extremely long transcripts. Increasing the library size threshold during the fragmentation step may improve the possibility of discovering longer transcripts. Additionally, owing to limited number of individuals and tissues used in the present study, the whole transcriptome of this species could not be fully covered in the sequencing libraries [39,40]. Therefore, a large-scaled examining different tissue types and at different physiological state may comprehensively illustrate the transcriptional diversity of goat.

The reliability of RNA-seq data is relatively high. However, it provides gene expression levels in a very large scale. Moreover, in the data analysis procedure, errors might be introduced. In this case, the accuracy of RNA-sequencing was further validated by qPCR to ensure that the sequencing data accurately captured the gene expression profiles of tissues or individuals. Quality control of the raw reads obtained in the present study showed that the quality of most bases of each sample was higher than Q_phred_ = 20 (Q20). Moreover, over 90% of the clean reads were successfully mapped to the goat genome, which is similar to other reported studies on domestic animals. However, not all reads obtained in this study were successfully mapped or annotated in the genome were annotated to functional genes, owing to GC content of the RNA, cell types, and imperfect genome annotation. Therefore, further improvements in the goat reference genome are necessary to increase the number of reads that can be annotated. In the present study, verification of the RNA sequencing data by qPCR, showed that the RNA sequencing data were reliable for depicting the transcriptome of LD and BF.

In the present study, genes regulating muscle development and meat quality were identified using hybrid sequencing technology. Meat quality has always been an important trait to consumers and meat producers. The quality traits of fresh meat are categorized on the basis of major intrinsic and extrinsic factors. Appearance quality traits generally include meat color, water-holding capacity, texture, and intramuscular fat (IMF) content [41]. OxyMb oxidation is the predominant determinant of meat color, which means that the higher the myoglobin (Mb) content in type I (slow-twitch) muscle fibers the higher the intensity of the red color [42]. In the present study, color analysis showed that the intensity of red color of BF muscle was higher than that of the LD muscle, indicating that the BF muscle should have more type I muscle fibers than the LD muscle. Tropomyosin 1/2/3 (encoded by TPM1/2/3 genes) is a member of the tropomyosin family, which is a highly conserved and widely distributed actin-binding protein [43]. The relative amounts of TPM1 are higher in fast-twitch (type II) fibers than in slow-twitch fibers in skeletal muscles, whereas TPM2 and TPM3 have a reverse expression pattern [44]. In the present study, TPM1 was highly expressed in LD, whereas TPM2 and TPM3 were highly expressed in BF. Other genes that indicated fast/slow fiber ratios, such as TNNC1 [45], MYL2 [46], TNNT1 [47], and TNNI1 [48], were also identified in the present study. They are all slow fiber-associated genes and were highly expressed in the BF muscles in comparison with in the LD muscles, indicating that the LD of the goats had a higher ratio of fast fibers than slow fibers; however, this should be verified through phenotyping. Studies have shown that BF is tougher than LD muscles [12]. In the present study, shear force analysis revealed that the BF muscle was tougher than the LD muscle, indicating that meats containing more slow fibers are tougher than meat containing more fast fibers. Nonetheless, it should be noted that the sample size of this study was limited, which might not reflect the phenotypes of large samples.

Marbling texture mainly refers to the IMF content of the meat, which affects flavor, juiciness, and tenderness. IMF content tends to increase with aging when the main process of muscle growth is complete. IMF biosynthesis is an adipogenesis process in muscle fibers. In the present study, several genes and isoforms related to muscle development and lipid metabolism were expressed in the LD and BF muscles of the goats, among which WNT5B, ADIG, and LPL were found to be differentially expressed in LD and BF muscles. WNT5B, ADIG, and LPL play important roles in adipose tissue development and lipid metabolism in white adipose tissue. WNT5B has been shown to have controversial roles in adipogenesis [49]. WNT5B activates adipocyte differentiation by promoting the expression of adipocyte markers and by relieving WNT3A-suppressed adipogenesis [50]. Conversely, WNT5A, which is closely related to WNT5B, inhibits adipogenesis in rat stromal vascular cells [51]. *ADIG* is a novel transcription factor involved in adipogenic differentiation. ADIG induces adipogenic differentiation of bovine myoblasts and 3T3-L1 pre-adipocyte cell lines [52]. However, another study found that ADIG had no effect on lipid accumulation [53]. LPL, as a hydrolytic enzyme, has dual functions as a triglyceride hydrolase and ligand/bridging factor for receptor-mediated lipoprotein uptake [54]. Previous research on WNT5B, ADIG, and LPL mainly focused on adipose tissue development except IMF; therefore, further studies should focus on their role in IMF formation.

The most abundant gene in the present sequencing data was CKM (creatine kinase, M-type). CKM protein reversibly catalyzes the transfer of phosphate between ATP and creatine phosphate, playing a central role in energy transduction in skeletal muscles and other tissues with fluctuating energy demands [55]. The high expression of CKM is a characteristic of skeletal muscles, such as LD and BF, which are closely involved in energy mobilization. The myosin light chain, phosphorylatable, fast skeletal muscle (encoded by MYLPF), is a cytoskeletal gene. This protein is an integral part of the myosin light-chain structure of the skeletal muscles. Previous studies have indicated that MYLPF expression decreases with increasing age in mice [56]. However, MYLPF was still highly expressed in eight samples from 10-month-old goats in the present study. However, the function and expression pattern of MYLPF in goats require further investigation. Myosin light chain 1 (MYL1) is another highly but not differentially expressed gene in our sequencing data. Inhibiting MYL1 expression delays myoblast differentiation in vitro [57]. The higher expression of MYL1 in LD muscle in the present study might indicate that LD muscle has a higher active differentiation status than BF. Actin alpha 1 (ACTA1) and skeletal muscle were also highly expressed in libraries, whereas actin Alpha 2 (ACTA2) and smooth muscle had much lower expression (approximately 1000 FPKM per sample) in the present study. Their encoded products belong to the actin family of proteins, which are constructed for cell motility, integrity, and structure. ACTA2 is mainly expressed in smooth muscles instead of skeletal muscles. Further investigation of the role of ACTA2 in skeletal muscle is required.

## 5. Conclusions

In the present study, second- and third-generation sequencing technologies were used to identify genes regulating meat quality and muscle development in goats. The meat quality and transcriptome of two types of skeletal muscle were compared. There was a total of 231 DEGs, in which 45 were novel. Additionally, 1173 isoforms, including 642 novel isoforms were found to be differentially expressed. Functional annotation and pathway analysis of the DEGs and DEIs revealed that some of them were enriched to multiple muscle development pathways and lipid metabolism, which determining the meat yield and quality. However, further studies are needed to comprehensively examine the functional roles of the novel isoforms identified in this study using large-scale gene function validation technologies such as CRISPR-CAS9.

## Figures and Tables

**Table 1:** Meat quality of longissimus dorsi and biceps femoris muscles in goats used in this study.

Item	Muscle Type		*p*-Value
	LD	BF	
pH45 min	6.71 ± 0.13	6.94 ± 0.13	0.26
pH24 h	5.72 ± 0.06	5.83 ± 0.08	0.28
Lightness, L	35.35 ± 1.59	35.23 ± 1.46	0.96
Redness, a	11.63 ± 0.75 ^b^	14.08 ± 0.41 ^a^	0.03
Yellowness, b	1.28 ± 0.43	0.53 ± 0.37	0.23
Water loss (%)	33.02 ± 2.12	28.14 ± 5.22	0.42
Water hold (%)	48.44 ± 1.98	46.33 ± 0.75	0.32
Shear force (N)	29.06 ± 2.54 ^b^	48.65 ± 7.2 ^a^	0.04
Marbling score	6.00 ± 0.00 ^a^	5.25 ± 0.25 ^b^	0.02

Data are shown as mean ± SEM, *n* = 4. ^a,b^. The values within a row with different superscripts are significantly different.

**Table 2:** Classification of circular consensus sequences in full-length sequencing.

Type of Reads	Library		
	1–2 kb	2–3 kb	3–6 kb
CCS ^1^	144,625	133,687	135,310
5′ reads	100,807	90,964	79,482
3′ reads	105,218	95,303	84,285
Poly-A reads	102,657	93,083	80,576
Filtered short reads	10,454	3043	6894
Non-full-length reads	46,175	50,806	60,436
Full-length reads	87,996	79,838	67,980
FLNC ^2^ reads	87,230	77,147	61,032
Average FLNC reads	1592	2604	2523

^1^ CCS, circular consensus sequences. ^2^ FLNC, full-length non-chimeric.

**Table 3:** Summary FLNC reads identified from full-length sequencing when aligned to reference genome.

Category	Pre-Correction	Post-Correction	Merge
Unmapped	2645 (1.17%)	1159 (0.51%)	1144 (0.51%)
Multiple-best	1467 (0.65%)	1506 (0.67%)	1333 (0.59%)
Low pid	14,116 (6.26%)	11,041 (4.90%)	9935 (4.41%)
High quality map	207,181 (91.91%)	211,703 (93.92%)	212,997 (94.49%)

**Table 4:** The loci and isoform annotation of the goat RefSeq and PacBio data.

Category	Annotation in RefSeq	Annotation in PacBio Sequences
Total Loci	22,570	18,491
Loci < 1 K	4284 (18.98%)	1214 (6.57%)
Loci 1–2 K	5770 (25.56%)	3254 (17.60%)
Loci 2–3 K	4401 (19.50%)	4637 (25.08%)
Loci ≥ 3 K	8115 (35.95%)	9386 (50.76%)
Total isoforms	46,472	45,574

**Table 5:** Differentially expressed genes identified from short-length sequencing that associated with muscle growth and tenderness by GO analysis.

Gene	BF (Mean FPKM)	LD (Mean FPKM)	log2 (Fold Change)	Enriched Terms
ANKRD1	158.10	447.93	1.49	1, 4, 6
ANKRD2	89.54	23.71	−1.88	1, 4, 8, 9
PITX1	13.10	0.48	−4.76	1, 7, 9
MYL2	11,534.42	1795.08	−2.66	2, 6, 10, 11
LOC106502520	3.71	1.10	−1.88	2, 6, 12, 13, 14
HOXD9	4.94	14.39	1.56	1
TPM1	8470.62	17,679.59	1.07	3, 6
FGF1	2.45	1.12	−1.12	3
FGF9	0.93	0.18	−2.38	3
PROX1	4.61	1.44	−1.66	5, 6
TNNT1	7761.57	3153.20	−1.29	12, 13
LOC102181869	4269.46	1419.53	−1.78	12, 13, 14
WNT5B	0.92	0.33	−1.45	15, 16, 19
ADIG	1.70	4.83	1.11	15, 16, 18, 19
LPL	90.98	28.05	−1.67	17, 21
LOC106502520	3.71	1.10	−1.88	22, 23, 24
LOC102181869	4269.46	1419.53	−1.78	22, 23, 24

1, skeletal muscle tissue development; 2, muscle fiber development; 3, muscle cell proliferation; 4, skeletal muscle cell differentiation; 5, skeletal myofibril assembly; 6, myofibril assembly; 7, myoblast fate commitment; 8, myoblast proliferation; 9, myoblast differentiation; 10, muscle cell fate commitment; 11, muscle cell fate specification; 12, transition between fast and slow fiber; 13, slow-twitch skeletal muscle fiber contraction; 14, regulation of slow-twitch skeletal muscle fiber contraction; 15, positive regulation of fat cell differentiation; 16, fat cell differentiation; 17, fatty acid biosynthetic process; 18, white fat cell differentiation; 19, regulation of fat cell differentiation; 20, adipose tissue development; 21, regulation of lipid storage; 22, BMP signaling pathway; 23, response to BMP; 24, cellular response to BMP stimulus.

## Data Availability

All original PacBio and Illumina sequencing data of this study is accessible with the following link: https://www.ncbi.nlm.nih.gov/bioproject/PRJNA755813/.

## Supplementary Materials

The following are available online at https://www.mdpi.com/article/10.3390/ani11102906/s1, Figure S1: KOG annotation of all novel isoforms. Figure S2: GO enrichment of all novel isoforms. Figure S3: KEGG pathway of all novel isoforms. Table S1: Composition level of diets (air-dry matter basis, %). Table S2: qPCR primers used in the present study. Table S3: Slaughter performance of goat individuals used in the present study. Table: S4. Reads yielding of short-read sequencing. Table S5: Short reads that mapped to the reference genome. Table S6: Short reads that mapped to the combined transcriptome. Table S7: LncRNAs identified in the full-length sequencing. Table S8: Statistics of the number of genes in different expression levels. Table S9: Statistics of the number of genes and isoforms in different expression levels. Table S10: Differentially expressed genes between BF and LD. Table S11: Differentially expressed isoforms between BF and LD. Table S12: GO enrichment of differentially expressed isoforms. Table S13: KEGG annotation of differentially expressed isoforms.
