# Supplementary material for: Hybrid Sequencing in Different Types of Goat Skeletal Muscles Reveals Genes Regulating Muscle Development and Meat Quality
Source: Animals (Basel). 2021 Oct 8;11(10):2906. doi: 10.3390/ani11102906 (PMC8532877; doi:10.3390/ani11102906)

Figure S1 KOG annotation of all novel isoforms

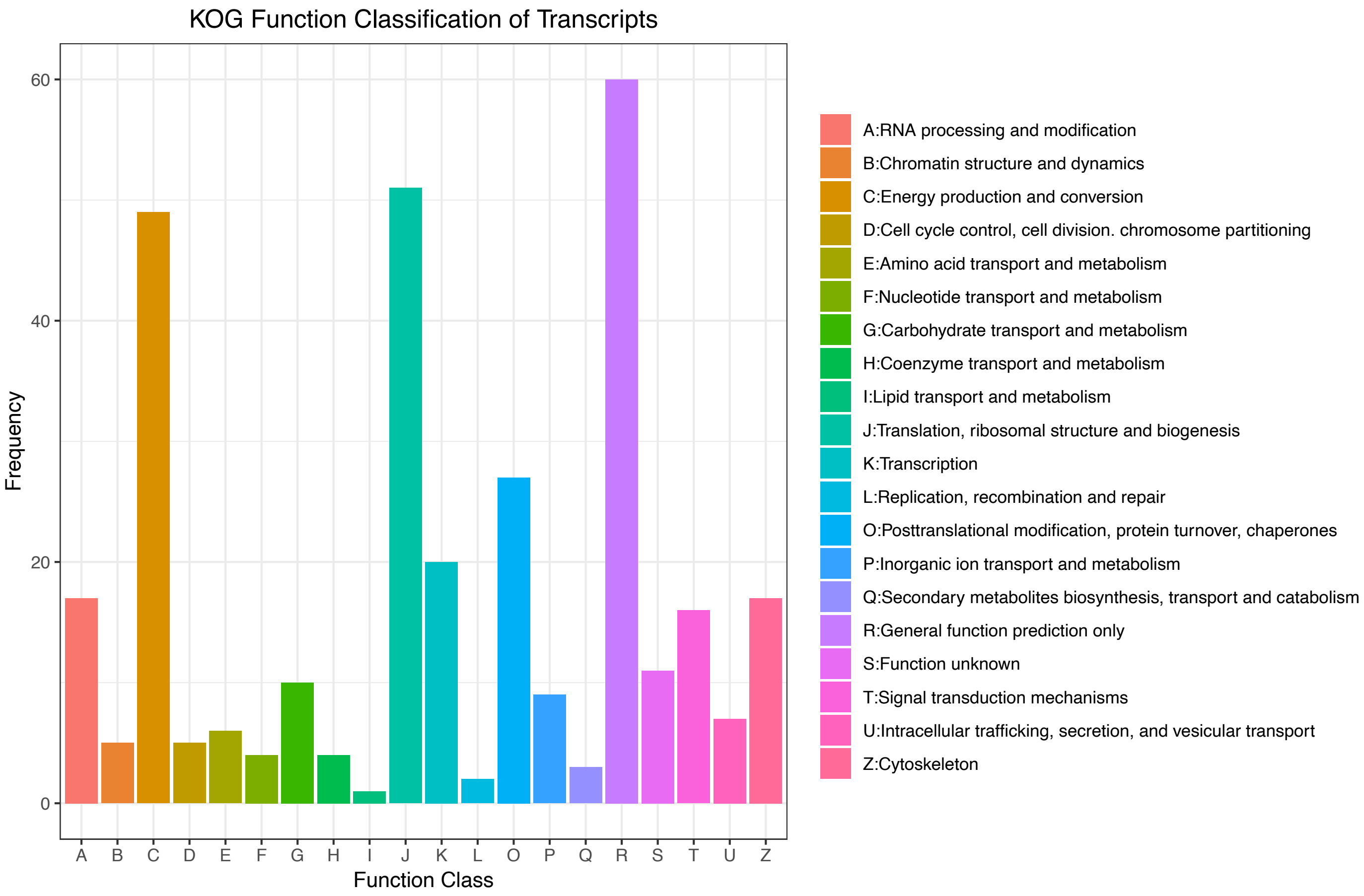

Figure S2 GO enrichment of all novel isoforms

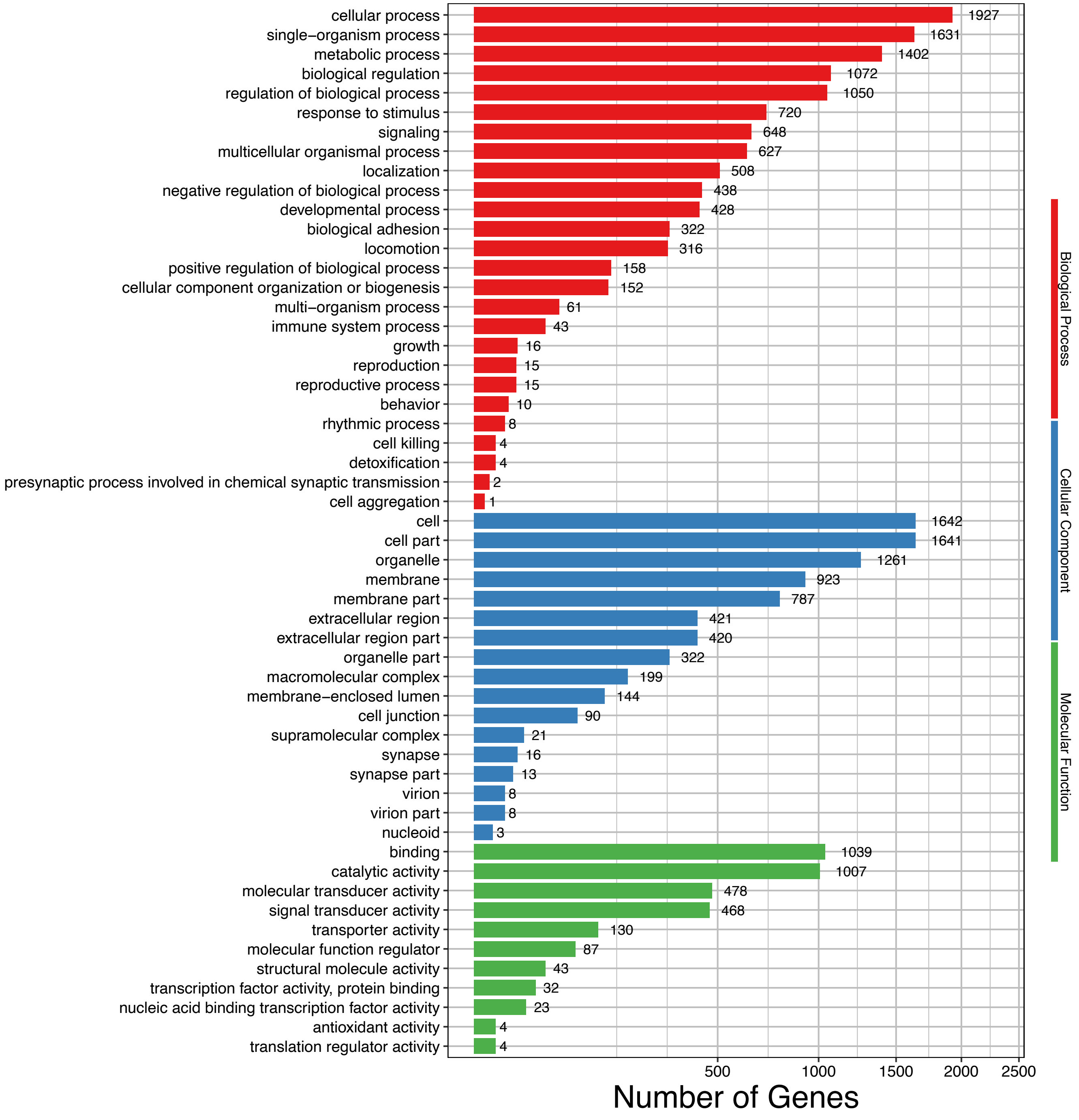

Figure S3 KEGG pathway of all novel isoforms

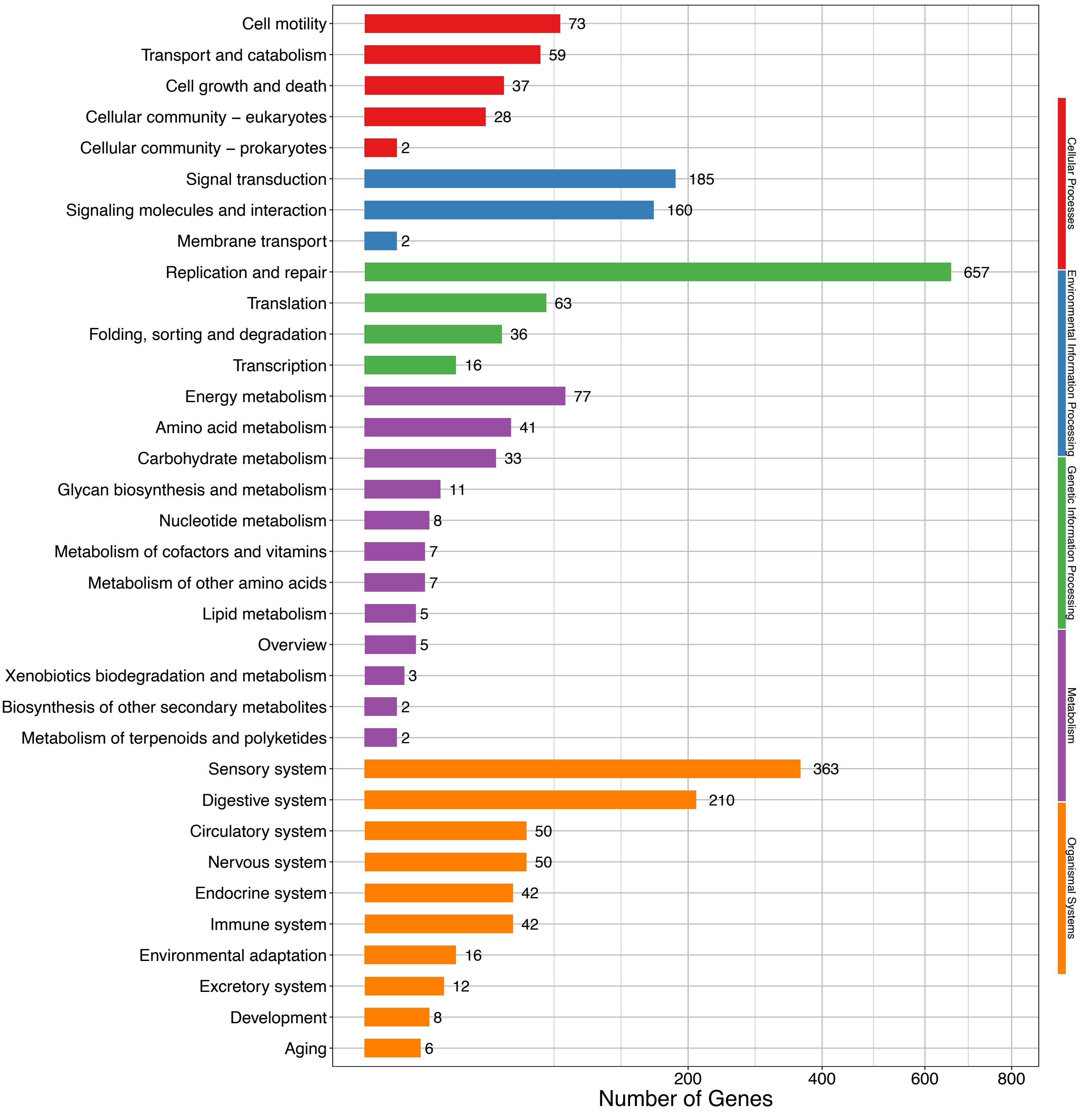

Supplement: Supplementary file 1 [file animals-11-02906-s001.zip › Supplementary figures.pdf]
